# Management of Pancreatic Cancer and Its Microenvironment: Potential Impact of Nano-Targeting

**DOI:** 10.3390/cancers14122879

**Published:** 2022-06-10

**Authors:** Nardeen Perko, Shaker A. Mousa

**Affiliations:** The Pharmaceutical Research Institute, Albany College of Pharmacy and Health Sciences, Rensselaer, NY 12144, USA; nardeen.perko@alumni.acphs.edu

**Keywords:** pancreatic ductal adenocarcinoma, molecular targeting, molecules signatures, nano-targeting, stellate cells, biotechnology, chemoresistance

## Abstract

**Simple Summary:**

The poor prognosis and survival rates associated with pancreatic cancer show that there is a clear unmet need for better disease management. The heterogeneity of the tumor and its microenvironment, including stroma and fibrosis, creates a challenge for current therapy. The pathogenesis of pancreatic cancer is mediated by several factors, such as severed communication between pancreatic stellate cells and stroma and the consequences of hypoxia-inducible factors that aid in the survival of the pancreatic tumor. Given the multiple limitations of molecular targeting, multiple functional nano-targeting offers a breakthrough in pancreatic cancer treatment through its ability to overcome the physical challenges posed by the tumor microenvironment, amongst many others.

**Abstract:**

Pancreatic ductal adenocarcinoma (PDAC) is rare and difficult to treat, making it a complicated diagnosis for every patient. These patients have a low survival rate along with a poor quality of life under current pancreatic cancer therapies that adversely affect healthy cells due to the lack of precise drug targeting. Additionally, chemoresistance and radioresistance are other key challenges in PDAC, which might be due in part to the lack of tumor-targeted delivery of sufficient levels of different chemotherapies because of their low therapeutic index. Thus, instead of leaving a trail of off-target damage when killing these cancer cells, it is best to find a way that targets them directly. More seriously, metastatic relapse often occurs after surgery, and therefore, achieving improved outcomes in the management of PDAC in the absence of strategies preventing metastasis is likely to be impossible. Nano-targeting of the tumor and its microenvironment has shown promise for treating various cancers, which might be a promising approach for PDAC. This review updates the advancements in treatment modalities for pancreatic cancer and highlights future directions that warrant further investigation to increase pancreatic patients’ overall survival.

## 1. Introduction

Pancreatic cancer is a disease with poor prognosis and survival rates. If pancreatic cancer develops from endocrine cells, the median rate of survival is around 27 months. On the other hand, if it develops from exocrine cells and results in pancreatic ductal adenocarcinoma (PDAC), then the survival rate is a few months to a year [1]. Thus far, in 2022, there have been 57,600 new pancreatic cancer cases diagnosed in the United States, including 47,050 deaths [2]. Furthermore, there are many risk factors linked to pancreatic cancer, including a family history of pancreatic cancer, obesity, cigarette smoking, and chronic pancreatitis. Certain genetic mutations that could also contribute to the diagnosis of pancreatic cancer include those associated with *BRCA1*, *BRCA2*, *PALB2*, and *ATM* genes [2,3]. Unfortunately, due to a lack of symptoms, pancreatic cancer often spreads before it is diagnosed; hence, it is a silent killer in its progression.

Many studies have examined different avenues for predicting a prognosis in these patients. Some prognostic values include the neutrophil-to-lymphocyte ratio, platelet-to-lymphocyte ratio, monocyte-to-lymphocyte ratio, and fibrinogen-to-albumin ratio [4]. All of these values are measured to detect changes in systemic inflammation, which is related to the process of metastasis. A systematic inflammation score exists to predict a patient’s overall survival and make more informed therapeutic intervention decisions [5]. In addition, cancer-specific survival for patients with cancer in the head of the pancreas is poor (overall 5-year survival rate of 2.5%), and they were more likely to develop a more progressive tumor stage than patients with pancreas body/tail cancer [6,7].

The treatment modality for pancreatic cancer is inclusive of surgical interventions, radiation, and chemotherapeutics. Despite having these three avenues for targeting the disease, treatment failure and relapse rates do not reflect well on the disease prognosis. Surgical resection takes place as the main tool for treatment, especially when the tumor is resectable, or as a palliative measure if the cancer is too widespread to be removed completely, indicated at certain disease checkpoints. However, following the surgery, the tumor comes back 80% of the time, and relapse occurs. Treatment options become even more limited after relapse, and the survival rate over three years is about 27% [8]. Therefore, much research and investigation still need to be conducted in pancreatic cancer due to the poor prognosis and survival rates. This review encapsulates the advances in pancreatic cancer treatments and the advancements that are aiming to increase patient survival rates.

## 2. The Pancreatic Cancer Microenvironment

To best understand any cancer, one must dive deep into understanding the nature of the tumor. Not all tumors are created equal, and some are much more challenging to eliminate than others. The pancreatic tumor is known for its intricate complexity, mainly due to its microenvironment. The pancreatic cancer microenvironment consists of malignant cells within an extracellular matrix, pancreatic stellate cells (PSCs), endothelial cells, and immune cells (Graphical Abstract Figure) [9].

Fortunately for the tumor, its microenvironment creates a challenge for therapeutic interventions to overcome, especially given its heterogeneity. Additionally, the tumor thrives in an oxygen-deficient microenvironment [1], making it difficult to sneak up on the tumor through the blood. The mechanism by which the pancreatic tumor thrives is not entirely understood, given the lack of angiogenesis that usually supports the growth of solid tumors [1]. The tumor is not supplied with nutrients in any known way, contributing to its complexity.

Many studies have examined PSCs to better understand their role in advancing pancreatic cancer. PSCs are highly involved in the pathogenesis of pancreatic cancer. In turn, studies are looking to find ways to suppress or inhibit interaction and communication between stellate cells and pancreatic cancer cells. Table 1 shows some of the different factors that researchers have studied to achieve this. The main role of PSCs is the expression of the α-smooth muscle actin protein involved in cytoskeletal processes, leading to significant amounts of extracellular protein production that creates that thick fibrotic barrier [10]. Through these studies and their investigation of unique mechanisms involved in pancreatic cancer, we can better understand what it is about pancreatic cancer that results in treatment challenges.

## 3. Treatment Resistance and Challenges

One of the biggest challenges with pancreatic cancer is relapse after surgery or chemotherapy, most likely because of cancer stem cells (CSCs), which are responsible for the renewal and tumor progression through metastasis [20].

Another contributor to relapse, but not necessarily to metastasis, is epithelial–mesenchymal transition (EMT). EMT gives CSCs the ability to have greater resistance to treatments such as 5-fluorouracil, cisplatin, gemcitabine, and epidermal growth factor receptor (EGFR) inhibitors. In its collaboration with CSCs, EMT’s mechanism has much investigation to go through before it is fully understood [1]. Another reason for treatment resistance is the hypoxic nature of the tumor in a heterogeneous manner. In-depth research in the area has identified that hypoxia modulates the PDAC’s tumor microenvironment through hypoxia-inducible factors (HIFs), which are responsible not only for tumor proliferation but also for chemo-radiotherapy and immunotherapy resistance [21]. Table 2 summarizes the roles that these HIFs play in the progression of pancreatic cancer [22].

## 4. Biotechnology Molecular Signatures

Many molecular signatures are associated with pancreatic cancer. Researchers obtain them by examining the activated genes, inactivated genes, genes associated with familial pancreatic cancer, and aberrantly methylated or hypermethylated genes. Table 3 shows several genes, whether activated or inactivated, that are thought to be the most important in the development of pancreatic cancer [23]. The mu-opioid receptor (MOR) and somatostatin receptor 2 (SSTR2) are unique receptors in PDAC or their malignant environment. Both receptors localize on the cellular membrane, and their activation leads to metastasis [24]. Despite having this wealth of molecular signatures, chemotherapies and current treatment guidelines cannot push patient survival rates past 25%.

## 5. Current Treatment Guidelines

The treatment guidelines focused on resectable or borderline resectable pancreatic cancer are different from those focused on locally advanced pancreatic cancer. There are four types of resectable pancreatic cancer treatments: neoadjuvant therapy, surgery, postoperative chemotherapy, and postoperative chemo-radiation options for pancreatic cancer. The role of chemotherapy in these treatments is either before or after surgery. Of course, during any stage of the disease, palliative care can always be considered [25].

## 6. Resectable or Borderline Resectable Pancreatic Cancer

There is evidence for several postoperative chemotherapy options, including FOLFIRINOX (oxaliplatin, leucovorin, irinotecan, and 5-FU), gemcitabine, capecitabine, and 5-FU monotherapy [2]. The Partenariat de Recherche en Oncologie Digestive (PRODIGE-24) trial studied a modified regimen of FOLFIRINOX in 493 patients and compared it with gemcitabine as adjuvant treatment for pancreatic cancer. Enrolled patients had resected PDAC. The study modified the FOLFIRINOX regimen and dosed it per square meter (SM): 85 mg/SM oxaliplatin, 150 mg/SM irinotecan, 400 mg/SM leucovorin, and 2400 mg/SM fluorouracil. Similarly, gemcitabine was dosed at 1000 mg/SM. The study results showed that the overall survival was significantly higher; it was greater than 10%, or 8.8 months, in patients who received the modified FOLFIRI-NOX treatment compared to gemcitabine. However, patients did experience greater adverse effects with FOLFIRINOX than with gemcitabine [26]. The adverse effects that had the greatest difference in the number of incidences (greater than 30%) include diarrhea (35.4%), paresthesia (52%), and sensory peripheral neuropathy (52.5%).

Other studies, such as the European Study Group for Pancreatic Cancer (ES-PAC-3), compared the use of gemcitabine alone versus fluorouracil with folinic acid. They learned that the median disease-free survival was 29.1 months for gemcitabine alone versus 23.0 months for fluorouracil with folinic acid, which demonstrated gemcitabine to be the superior agent [27]. In ESPAC-4, they compared gemcitabine monotherapy with gemcitabine and capecitabine dual therapy, and the dual therapy showed superior survival, with an increase of 3.5 months [28]. Therefore, the guideline-recommended chemotherapy treatment is useful for pancreatic patients. However, the disease prognosis is still very poor, and chemoresistance remains a problem.

## 7. Treatment-Associated Side Effects

There are many undesirable side effects experienced by pancreatic cancer patients who are treated with conventional chemotherapeutic agents. For example, adverse effects with gemcitabine include leukopenia, decreasing platelets, neutrophils with fever or infection, and non-hematologic toxicity greater than grade 3. The Metastatic Pancreatic Adenocarcinoma Clinical Trial study (MPACT), a phase III trial of nab-paclitaxel plus gemcitabine, demonstrated greater adverse effects than for gemcitabine alone as the treatment frequency lengthened, such as grade III peripheral neuropathy, anemia, neutropenia, and mucosal inflammation [29]. Toyama et al. studied the implications of biweekly adjuvant therapy with gemcitabine to determine whether it succeeded in prolonging overall survival and disease-free survival while at the same time avoiding the worsening of quality of life. They reported that adjuvant chemotherapy with biweekly gemcitabine was associated with better survival and fewer side effects in pancreatic cancer patients (median OS, 20.2 versus 11.9 months, *p* < 0.005). [30].

## 8. Treatment Failure

One of the most significant reasons for treatment failure is the extensive architecture of the stromal cells that have succeeded in creating a physical barrier through which chemotherapeutics such as gemcitabine cannot cross [31]. Therefore, much needs to be studied to better fathom the communication parameters between the stroma and the tumor cells. Approaches have been tried to affect the stromal architecture to cause permanent disruption of its extensive involvement in lowering treatment response [31]. However, despite these efforts, much is still not understood, and thus, the treatment protocol continues to be lacking.

Studies are currently going in two directions to increase the overall survival of pancreatic cancer patients. One route focuses on the extensive fibrous tissue and overcoming it so that treatments may become more effective. The other route tries to get past the stroma in innovative ways and arrive directly at the tumor. There is a third possible route that is a combination of these two routes.

Treatment failure is often due to chemoresistance in pancreatic cancer. Preclinical trials are studying new mechanisms to overcome this challenge, explicitly using nanotechnology. In the next few sections, we cover how nanotechnology can play a significant role in eliminating treatment failure.

## 9. Impact of Nanotechnology in Pancreatic Cancer

Nanotechnology is a new realm in medicine, and it has many properties that make it an impressive collaborator with today’s medicines. The introduction of nanomaterials’ physicochemical characteristics to medicine has had profound benefits. They can help enable novel therapies based on traditional therapeutics and overcome many limitations, such as physical barriers encapsulating tumors, as in pancreatic tumors. Currently utilized nano-shells can be composed of liposomal platforms, polymeric platforms, and other platforms that are nanocrystalline [4,32]. Nanotechnology used in medicine, called “nanomedicine”, has opened up many possibilities due to its functionality in enhancing traditional medicine’s capabilities.

Targeted therapy with nanoparticles has been inserted into treatment modalities, from cardiovascular treatments to oncology treatments. Due to the small size of nanoparticles, they can be suitable for crossing the blood-brain barrier (BBB), specifically nanodiamonds with conjugating potential [33]. Researchers are trying to formulate a conjugation between a drug that does not cross the BBB, such as amlodipine, and nanodiamonds that do cross it [34]. The nano’s ability to allow drugs to cross the BBB means it is a model that can be used for neurodegenerative diseases such as Alzheimer’s to reverse neuronal cell death [33]. Drugs can be loaded on the surface of a nanoparticle or inside a nanoparticle to allow for targeted therapy to reach pathways that otherwise may have been inaccessible. For example, there have been nanosystems models constructed to treat drug-resistant breast cancer patients. Lou et al. constructed a multilayered nanosystem composed of a poly core, liposome, and chitosan from the inside to the outside, respectively [34]. This nanocapsule design included three drugs within the core (doxorubicin, paclitaxel, and silybin) to target the CD44S receptor, an overexpressed biomarker in breast cancer. A xenograft animal model showed a fivefold decrease in tumor volume, resulting in tumor regression [34]. Therefore, to overcome the challenging cancer pathophysiology in pancreatic cancer, nanoparticles may be best suited when targeting multiple pathways to inhibit tumor progression.

### 9.1. Current Applications of Nanomedicine in Pancreatic Cancer

There is much that can be learned from the current use of nanotechnology in pancreatic cancer that can be applied elsewhere. For instance, targeted therapy using nanoparticles is being discussed more and more for the challenging pancreatic cancer tumor; however, it can be applied to other disease states for a number of reasons. Many drug therapies induce side effects due to their effect on healthy cells or cells neighboring the tumors, and these side effects are often deemed manageable in deference to the clinical efficacy of the treatment. However, nanotechnology introduces the idea that nano-targeted therapy can have the potential to deliver any drug, whether a chemotherapeutic drug or endocrine drug, exactly where it is meant to act, bypassing healthy cells, without allowing it to affect the patient’s quality of life. This can greatly increase adherence to treatment. Next, nanoparticles introduce the possibility of combining more than one drug and administering them at once instead of administering a number of tablets or capsules. This would greatly contribute to resolving the problem of polypharmacy, especially in the elderly population, which would benefit greatly from a reduced side effect profile.

### 9.2. Nano-Targeting vs. Molecular Targeting

Nano-targeting places itself at a tremendous clinical advantage due to its characteristics and capabilities. It translates into the potential for lowering drug administration frequency and delivering multiple drugs at once, thereby increasing patient adherence and minimizing side effects. Through therapeutic drug combinations, it also allows for the expression of synergistic effects and can overcome drug resistance, especially in oncology [32]. Molecular targeting focuses on finding a molecular signature, a gene or protein expression related to the cancer cell’s behavior and targeting it. However, molecular targeting and nano-targeting have advantages in different areas. Molecular targeting works well in disease states that do not have a barrier to the drug and molecular signature, including trastuzumab (Herceptin), which is approved to treat certain breast and stomach cancers that overexpress human epidermal growth factor receptor 2 protein (HER-2) [35]. In contrast, nano-targeting works best in areas where many limitations exist with molecular targeting, and a need exists to push treatment boundaries for diseases such as pancreatic cancer [36].

### 9.3. Nano-Targeting versus Drug Targeting

There are many nanoparticle-based delivery systems, including polymer–drug conjugates, block copolymers, mixed micelles, graft polymers, dendrimers, thermo-responsive polymers, pH-responsive polymers, ultrasound-responsive nanoemulsions, albumin, and inorganic nanoparticles. For example, it was shown that 3,3′-diindolylmethane (DIM) and ellagic acid (EA) separately had inferior suppression of 50–60% of the pancreatic tumor compared to the nanoformulation combination of DIM and EA [37,38]. The combination of the two in a nano-shell had greater tumor angiogenesis suppression, as proven by the chick chorioallantoic membrane (CAM) tumor implant model. The use of DIM in this nanoformulation demonstrates the immense benefit of using a nanoformulation because it improves the chemical and physical properties of the insoluble DIM to assist in its delivery. Cellular uptake of the cancer drug was increased, stability was improved, and release was sustained. This demonstrates the significant potential for improvement in the suppression of the pancreatic tumor with a nanoformulated paclitaxel and gemcitabine combination in comparison with the parent drugs [39]. Hence, nanoformulations have a bright future in improving the treatment of pancreatic cancer, as has been demonstrated with natural products.

One of the more commonly targeted proteins in pancreatic cancer is the αvβ3 integrin. This integrin is overly expressed on pancreatic tumor cells. For example, a rationally designed novel therapeutic protein known as ProAgio was designed to target this integrin specifically [38]. Studies of ProAgio have shown that it causes apoptosis of pancreatic cancer cells that express αvβ3, which enhanced the efficacy of gemcitabine therapy. Therefore, αvβ3 is one of the effective targets in pancreatic cancer due to its increased expression in tumorigenesis, whereas other integrins such as αvβ6 do not contribute positively to PDAC.

Madamsetty et al. developed new tumor-targeted dual intervention-oriented drug-encapsulated liposomes (DIODE) for study in PANC-1 cells. These liposomes were loaded with gemcitabine, erlotinib, paclitaxel, and XL-184 for study in vivo and in vitro in preclinical trials. The study measured tumor growth for a single mouse per treatment arm to identify the best treatment regimen. The drugs in the nanoliposome had greater targeting in comparison with the drug alone in the control group in terms of tumor growth suppression, prolonging 1-year survival by 23% [39].

## 10. Current Role of Nanotechnology in Pancreatic Cancer

Nab-paclitaxel is a nanoparticle albumin-bound formulation of paclitaxel using passive targeting, which has already shown greater efficacy than the previous standard of care treatments by extending patients’ survival by five to six months. This points to new nanoparticle formulations that are not albumin-bound but could have greater efficacy, a higher overall survival rate, and a lower side effect profile using active targeting strategies. Such formulations could combine gemcitabine and paclitaxel in a nano-shell and target the delivery to the tumor, leaving healthy cells untouched while administering a higher dose compared to chemotherapeutic agents alone. However, the disease state’s challenges can be combatted by composing a nano-shell that carries these drugs directly to the site of action to minimize any unwanted side effects and warrant the administration of higher doses.

### Locally Advanced Pancreatic Cancer

The treatment difference between therapies for resectable or borderline resectable and locally advanced pancreatic cancer is the addition of two drugs (nab-paclitaxel and erlotinib) to help with advanced, difficult-to-treat stages. Nanoformulations can extend the use of these drugs, which have limited success rates alone [2]. Many studies have demonstrated the superiority of the nab-paclitaxel and gemcitabine combination versus using gemcitabine alone to treat advanced pancreatic cancer, as described next.

The marker used to detect this superior efficacy is carbohydrate antigen 19-9 (CA19-9), which has some limitations but is the most-studied tumor marker that provides a mortality outlook in patients with pancreatic cancer. In the albumin-bound paclitaxel (ABI-007) study, the decrease in CA19-9 was clinically significant in overall survival for CA19-9, with decreases of ≥20% and 60%, but not 90%. The overall survival median for the combination therapy versus gemcitabine alone was 11.1 months versus 9 months [29]. Another study also found that nab-paclitaxel and gemcitabine provided longer overall survival than gemcitabine alone, 8.7 versus 6.6 months, respectively [40]. In addition, the nab-paclitaxel and gemcitabine combination are especially effective after FOLFIRINOX failure [41]. Thus, nab-paclitaxel brings some hope to patients with advanced stages of pancreatic cancer, and thus, it is added when a patient has locally advanced pancreatic cancer or other chemotherapies have failed.

## 11. Comparison among Targeting Approaches

### 11.1. mTOR Pathway

mTOR has mechanisms in place that maintain the properties of cancer stem cells. It mediates the signaling of the phosphoinositide 3-kinase/Akt pathway, which has activity in pancreatic cancer. However, of the five patients enrolled in the study, two died within a month due to rapid disease progression and hemorrhagic stroke [42]. Another study attempted to combine gemcitabine with temsirolimus, an mTOR inhibitor, to determine if they have synergy in enhancing overall survival. However, median progression-free survival was 2.69 months, median overall survival was 4.95 months, and the 6-month progression-free survival rate was 30.9% [43]. A study by Kordes et al. paired 5-fluorouracil with everolimus, another mTOR inhibitor. This study was designed following an observation that the combination of everolimus with capecitabine had potential activity in patients with pancreatic cancer. However, the adverse effect profile was significant for hyperglycemia (45%), hand-foot syndrome (16%), grade 1/11 anemia (61%), fatigue (55%), and rash (65%) [44]. Therefore, despite the combination of everolimus with capecitabine being moderately active with a manageable toxicity profile, there should be further investigation before this can be implemented in treatment modalities.

### 11.2. Hedgehog Signaling

The hedgehog signaling pathway plays a role in the development of adult tissue and can facilitate the dysregulation of cancer, hence making it a viable target of cancer therapy. However, this signaling pathway is complex and intricately layered, making drug targeting challenging. Inhibitors have been developed, such as RNA molecules, that have shown great promise in antagonizing this signaling pathway [45]. Vismodgib, an inhibitor of the hedgehog pathway, has been studied in combination with gemcitabine in patients with metastatic pancreatic cancer to exploit a potential synergistic effect and improve drug targeting. However, this has not shown significant changes in overall survival in pancreatic cancer patients [46]. Another oral inhibitor known as IPI-926 was studied alongside FOLFIRINOX to determine whether this combination could increase chemotherapeutic delivery by eliminating the challenge posed by the surrounding stroma. Although this study showed some feasibility in the initial stages, the phase II trial was stopped due to the lack of benefit of this combination, which was demonstrated in a separate phase II trial of IPI-926 plus gemcitabine. In mouse models, IPI-926 was shown to deplete tumor-associated stromal tissue and increase the intra-tumoral mean vessel density, which illustrated prolonged survival. However, when IPI-926 was administered in phase II trials, patients with metastatic pancreatic cancer had a shorter median survival time and more rapid disease progression in comparison with placebo [47]. Therefore, there remains a need for studies to be designed to explore nanoparticle delivery vehicles to better target these drugs to yield greater success than drug targeting alone [45].

### 11.3. Kinases

A mutated driver of many cancers, including pancreatic cancer, is KRAS, which is currently being studied as a target for therapies. Inhibiting this pathway has shown much promise due to the body’s ability to keep its downstream pathway in check through a different mechanism, specifically the RAF-MEK-ERK cascade [48]. Therefore, a novel approach to enhancing the effects of KRAS inhibition is the composition of a synergistic relationship with BI-3406, an inhibitor of SOS1. As a KRAS activator, SOS1 can potentiate the effects of KRAS in various cancers; therefore, its inhibitor along with a KRAS inhibitor should cause greater inhibition of the cascade effects than KRAS alone. Further studies are warranted to better understand the degree of clinical significance that this adds to the current treatment of pancreatic cancer [48].

### 11.4. Innovative Nano-Targeting Approaches

New ideas are circulating in terms of how to target the delivery of chemotherapeutics innovatively and differently than what has been done before. One study suggests studying the combination of nanoparticle drug delivery systems with intra-arterial delivery systems [49]. Intra-arterial delivery has been studied separately from nano-targeted delivery but bringing the two together could provide an innovative answer for pancreatic cancer. In a recent study using an orthotopic mouse model of pancreatic cancer, gemcitabine was delivered intra-arterially, and this suppressed tumor growth compared to dosing intravenously, which could result in poor patient outcomes due to significant toxicity. Intra-arterial delivery, however, can deliver a chemotherapeutic directly to the pancreas [49].

There are presently several clinical trials that are testing inventive additions to the current therapeutic regimens available for pancreatic cancer; some of the ongoing nano-trials in pancreatic cancer are highlighted in Table 4. A phase II trial is now looking at combining losartan with FOLFIRINOX and nivolumab [50]. Another study in phase I is assessing the use of warfarin in pancreatic cancer patients due to its effects on AXL pathways, which are found to have great implications in metastasis of pancreatic cancer [51]. Therefore, it is evident that a wealth of knowledge is on the horizon for the management of pancreatic cancer.

## 12. Future Directions

Future directions for improved pancreatic cancer management are in progress despite the low survival rate. This is especially true with the possibilities opened by nanotechnology, molecular targeting strategies, and the combination thereof. There is no question that the efforts of these innovative and preclinical trials, which are researching different nanoformulations, and the questions they address will provide a scope of focus for pancreatic clinical cancer research. Table 5 highlights ongoing preclinical investigations for nano-targeted strategies in pancreatic cancer research [58,59,60,61,62,63].

## 13. Conclusions

Pancreatic cancer is one of the most challenging disease states under the cancer umbrella, with researchers studying multiple mechanisms to find treatments that advance treatment modalities. Current treatments have failed to combat the pancreatic tumor’s nature and its stroma, which acts as a strong barrier and hypoxic microenvironment. Many pathways communicate with one another in pancreatic cancer in ways that we have yet to understand in order to design a more effective therapy. In addition, comparative targeting approaches such as mTOR, hedgehog, and kinases have had conflicting results in their clinical efficacy contribution when paired with current chemotherapeutic agents.

On the flip side, there is hope in current research and investigational efforts. Many preclinical and clinical trials are in place to push the boundaries of the current body of knowledge accumulated for pancreatic cancer. Some of these studies are testing various nanoformulations using current treatment regimens. Many doors will open through nanotechnology, especially in improving the efficacy and safety of existing chemotherapy and new molecular targets. The targeting potentials of nanotechnology will be instrumental in introducing new dimensions for improving pancreatic cancer management.

## Figures and Tables

**Table 1:** Summary of factors that, once inhibited, aid in damaging or severing communication between pancreatic stellate cells and stroma [11,12,13,14,15,16,17,18].

Factor	Involvement in Pancreatic Cancer	Reference
CXCL12	Averts cytotoxic T cells from penetrating the tumor and killing cancer cells	[12]
COL1A1	Involved in collagen deposition and plays a vital part in PDAC’s aggressive behavior	[13]
Survivin	Encourages apoptosis and augments gemcitabine sensitivity	[13]
CD51	Aids in stromal formation of pancreatic cancer and enhances tumor malignancy	[17]
Autophagy	Aids pancreatic cancer cells in producing extracellular matrix molecules and IL-6, which is associated with shorter survival and cancer recurrence	[16]
Hic-5	Enhances proliferation, decreases apoptosis, and increases invasion and migration of pancreatic cancer cells	[15]
Receptor Tyrosine Kinases (PDGFRβ and MET)	May play a role in modulating interactions between pancreatic cancer cells and pancreatic stellate cells	[14]
ERK 1/2	Involved in cancer stromal interactions and metastasis	[11]
Endo180	Enhances invasion abilities of pancreatic stem cells via phosphorylation of myosin light chain 2 (MLC2)	[19]
OPN	A phosphorylated glycoprotein overexpressed and secreted in activated pancreatic stem cells driven by hypoxia	[18]
FOXM1	Upregulated and induces malignant phenotypes of pancreatic cells	[18]

CXCL12: C-X-C motif chemokine ligand 12; COL1A1: collagen type I alpha 1 chain; CD51: integrin αV; Hic-5: hydrogen peroxide-inducible clone 5; PDGFRβ: platelet-derived growth factor receptor-β; MET: tyrosine protein kinase; ERK: extracellular signal-regulated kinases; Endo180: CD280, MRC2, urokinase-type plasminogen activator receptor-associated protein; OPN: ostepontin; FOXM1: forkhead box protein M1.

**Table 2:** Summary of hypoxia-inducible factors and the resultant effects that aid in the survival of the pancreatic tumor.

Hypoxia-Inducible Factor (HIF)	Effects’ References
HIF-1α	- Upregulates HLA-G [21,22] (Major Histocompatibility Complex, Class I, G) transcription - Increases autophagy - Increases glucose supply - Promotes cancer proliferation via interactions with notch signaling - Mediates NFκβ pathways, increasing N-cadherin, resulting in transendothelial migration into blood vessels
HIF-2α	- Promotes cancer cell generation in the cell cycle [21,22] via stabilization of MYC proto-oncogene - Upregulates survivin production, providing resistance to apoptosis by trial - Increases DNA repair mechanisms
HIF-3α	Inhibits other HIF complexes [21,22]

**Table 3:** Summary of molecular signatures, both activated and inactivated, that are thought to be important in the pathogenesis of pancreatic cancer besides others listed in Table 2.

Activated Genes	Inactivated Genes’ References
Kirsten rat sarcoma viral oncogene homolog (*KRAS2*)	Cyclin-dependent kinase [23,24] inhibitor 2A (*CDKN2A/P16*)
V-akt murine thymoma viral oncogene homolog 2 (*AKT2*)	Tumor protein p53 (*TP53*) [23,24]
V-raf murine sarcoma viral oncogene homolog B1 (*BRAF*)	Mitogen-activated protein [23,24] kinase 4 (*SMAD4/DPC4*)
	Transforming growth factor B receptor II (*TGFBR2*)
	Serine/threonine kinase 11 (*STK11/LKB1*) Breast cancer 2, early onset (*BRCA2*)
	MutL homolog 1 (*MLH1*)
	Mothers against decapentaplegic drosophila, homolog of, 4 (*SMAD4/DPC4*)

**Table 4:** Summary of selected ongoing nano-trials in pancreatic cancer.

Phase	Intervention/Treatment	Aim of Study	Reference
Early phase I	Warfarin given at 5 different doses if warfarin is assigned (as a placebo), ranging from 1 mg to 5 mg.	To confirm evidence that AXL activation is critical for tumorigenesis and metastasis of pancreatic cancer.	[51]
Phase II	Single-armed study of locally advanced pancreatic cancer, using CPI-613 with modified FOLFIRINOX combination.	To determine if CPI-613 increases overall survival in combination with modified FOLFIRINOX.	[52]
Phase I	NBTXR3 (hafnium oxide nanoparticles), when activated by radiation therapy, may cause targeted destruction of cancer cells to treat borderline resectable or advanced pancreatic cancer.	To determine the recommended phase II dose of NBTXR3 activated by radiation therapy.	[53]
Phase Ib/II	Phase Ib-selective inhibitor of nuclear transport (SINE) selinexor (KPT-330), gemcitabine, and nab-paclitaxel-and phase II-selinexor and gemcitabine-in patients with metastatic pancreatic cancer.	To determine the efficacy of selinexor, gemcitabine, and nab-paclitaxel in stopping the growth of tumor cells either by killing cells, stopping cell division, or stopping metastasis.	[54]
Phase Ib/II	IMX-110, a nanoparticle encapsulating a Stat3/NF-kβ/poly-tyrosine kinase inhibitor and low-dose doxorubicin.	To assess safety, tolerability, and pharmacokinetics for recommended phase II dose of IMX-110.	[55]
Phase 3	Methylnaltrexone bromide (MNTX) administered at 450 mg once daily by mouth. Treatment continues until participant’s death, early withdrawal from study, or study completion at day 168.	To evaluate safety and efficacy of oral MNTX tablets.	[56]
Phase 2	SGT-53, a complex of cationic liposome encapsulating a normal human wild-type p53 DNA sequence in plasmid backbone, which has been shown to deliver p53 cDNA to tumor cells. Used alongside nab-paclitaxel.	To evaluate safety, tolerability, toxicity, and efficacy (progression-free survival at 5.5 months) of this combination therapy	[57]

**Table 5:** Tumor-associated antigens for the delivery of therapeutic payloads in PDAC.

TAA	NP Carrier	Surface Modifier	Payload	Application	Ref.
MUC1	PLGA	MUC1 Ab (TAB004)	Paclitaxel	Ab-mediated chemo Delivery	[58]
MUC4	CPG and CPTEG	MUC4β protein	MUC4β	Immunotherapy	[59]
	Liposome	CA19-9 Ab	Doxorubicin	Ab-mediated chemo delivery	[60]
KRAS G12D	Glycol-Poly-L-lysine copolymer	Human scFv (CD44v6) Ab	siRNA	siRNA delivery	[61]
EGFR	Liposome	EGFR (Cetuximab) Ab	Benzoporphyrin derivative	photoacoustic imaging, PDT	[62]
	Micelle		Gemcitabine + Olaparib	Ab-mediated chemo Delivery	[63]
TF	Liposome	Anti-TF Ab	Gemcitabine + Paclitaxel	Ab-mediated chemo Delivery	[64]
Thyrointegrin *α*v*β*3	PLGA	Integrin *α*v*β*3	Chemotherapies	Small Molecule-mediated	[65,66,67]

Footnote: The human mucin gene *MUC4* is overexpressed in pancreatic cancer and cancer cell lines while remaining undetectable in the normal pancreas. Cancer antigen-19-9 is a tumor marker used primarily in the management of pancreatic cancer. KRAS G12D is the most prevalent mutation form that drives the most prevalent type of pancreatic cancer.

## Data Availability

Not applicable.
